# Understanding importance of clinical biomarkers for diagnosis of anxiety disorders using machine learning models

**DOI:** 10.1371/journal.pone.0251365

**Published:** 2021-05-10

**Authors:** Amita Sharma, Willem J. M. I. Verbeke

**Affiliations:** 1 Erasmus University, Rotterdam, Netherlands; 2 Department of Operations Research & Quantitative Analysis, Institute of Agri-Business Management, Swami Keshwanand Rajasthan Agricultural University, Bikaner, Rajasthan, India; National Institutes of Health, UNITED STATES

## Abstract

Anxiety disorders are a group of mental illnesses that cause constant and overwhelming feelings of anxiety and fear. Excessive anxiety can make an individual avoid work, school, family get-togethers, and other social situations that in turn might amplify these symptoms. According to the World Health Organization (WHO), one in thirteen persons globally suffers from anxiety. It is high time to understand the roles of various clinical biomarker measures that can diagnose the types of anxiety disorders. In this study, we apply machine learning (ML) techniques to understand the importance of a set of biomarkers with four types of anxiety disorders—Generalized Anxiety Disorder (GAD), Agoraphobia (AP), Social Anxiety Disorder (SAD) and Panic Disorder (PD). We used several machine learning models and extracted the variable importance contributing to a type of anxiety disorder. The study uses a sample of 11,081 Dutch citizens’ data collected by the Lifelines, Netherlands. The results show that there are significant and low correlations among GAD, AP, PD and SAD and we extracted the variable importance hierarchy of biomarkers with respect to each type of anxiety disorder which will be helpful in designing the experimental setup for clinical trials related to influence of biomarkers on type of anxiety disorder.

## Introduction

Anxiety disorders are a well-known phenomenon, afflicting about 20% of the US population, and come in different phenotypes, such as social phobia or general anxiety [1]. Anxiety disorders are a group of mental illnesses that cause constant and overwhelming feelings of anxiety and fear. Excessive anxiety can make an individual avoid work, school, family get-togethers, and other social situations that might trigger or worsen the symptoms. Primarily, four anxiety disorders–Generalized Anxiety Disorder (GAD), Panic Disorder (PD), Social Anxiety Disorder (SAD) and Agoraphobia (AP) are the focus in this study conducted on Lifelines database (see later for a description of this data base in The Netherlands). Diagnosis of a particular type of anxiety disorder is very important and it is carried out by a set of measures that might include physical symptoms and social isolation as screening measures. Physical symptoms include e.g. chest pain, shortness of breath, overeating, sleep or remain awakened, showing disinterest, avoid going out, abusive use of alcohol and drugs. Social isolation such as avoiding social gatherings with many friends or not willing to go to school or work are other major signs of anxiety disorder.

Comorbidity with psychiatric disorders is common, especially with major depressive disorder, but others include multiple anxiety disorders (PD, SAD, post-traumatic stress disorder [PTSD], GAD), and dementia [2]. Diagnosis of anxiety disorders can be further complicated if patients do not report symptoms associated with anxiety to their physician. This situation can occur when a patient feels there is a negative stigma associated with a mental disorder diagnosis [3]. Challenges in screening suspects of anxiety disorders are many. GAD and Major Depressive Disorder (MDD) are highly comorbid. Zbozinek et al. [4] suggested that comorbidity of GAD and MDD is strongly influenced by diagnostic overlap.

Pintelas et al. [5] reviewed the 16 studies which implemented machine learning (ML) techniques to predict the specific type of anxiety disorders. In these studies, none of the study worked on classification of a subject having a particular type of anxiety disorder. Most relevant for this paper, many of these studies used no biomarkers data or a few biomarkers data. Majority of the studies dealt with comorbidity of anxiety disorders with other disorders like MDD.

The objective of this study is to find the variable importance hierarchy of biomarkers for anxiety disorder and a particular type of anxiety disorder, not the prediction of one particular anxiety disorder. We assume that there should be an underlying complex relationship between biomarkers and a type of anxiety disorder. To explore this, we are using univariate and multivariate models—Logistic Regression, Random Forest, Support Vector Machine, and Neural Net with default settings (see S2 Appendix) to obtain an aggregate list of variable importance of 28 biomarkers for each type of selected anxiety disorder.

This study is useful for the researchers who aim to conduct clinical trial-based studies wherein a particular set of biomarkers is used as independent variables for predicting or associating a particular type of anxiety disorder. This study will be helpful in pre-screening and choosing a set of biomarkers from a list of 28 biomarkers. Briefly, this research also explores the association among the four anxiety disorders–GAD, AP, SAD and PD which were more prevalent in Lifelines database. We believe that experimental studies for identifying a particular type of anxiety disorder based on biomarkers’ measurement are essential to establish etiology. Our study helps in prioritizing the biomarkers for such experimental studies.

This study neither aims to predict the type of anxiety disorders, nor comorbidity with other disorders like MDD, nor comorbidity cases among four disorders and nor screening anxiety disorder patients from healthy subjects based on ML as this study applies ML models to a small sample size of 11081 respondents. For ML models, the sample size 11081 is comparatively very small dataset. To avoid model overfitting, we applied simple ML models with default settings and didn’t apply high level machine learning ensembles.

### What is anxiety disorder?

Anxiety disorder is a normal emotion that is triggered by the brain in response to stress. It alerts the individual from potential dangers. Everyone feels anxious often. The occasional occurrence of anxiety is normal but if it is excessive, constant and overwhelming due to a group of mental illnesses, it manifests into anxiety disorders [6].

Below, we provide a brief description of the four anxiety disorders, as defined according to the American Psychiatric Association [7]: A) “GAD involves persistent and excessive worry that interferes with daily activities. This ongoing worry and tension may be accompanied by physical symptoms, such as restlessness, feeling on edge or easily fatigued, difficulty concentrating, muscle tension or problems sleeping. Often the worries focus on everyday things such as job responsibilities, family health or minor matters such as chores, car repairs, or appointments”. B) AP is “the fear of being in situations where escape may be difficult or embarrassing, or help might not be available in the event of panic symptoms. The fear is out of proportion to the actual situation and lasts generally six months or more and causes problems in functioning. A person with AP experiences this fear in two or more of the situations like using public transportation, being in open spaces, being in enclosed places, standing in line or being in a crowd, being outside the home alone. The individual actively avoids the situation, requires a companion or endures with intense fear or anxiety”. C) In PD, “the core symptom is recurrent panic attacks, an overwhelming combination of physical and psychological distress. During an attack several symptoms like palpitations, pounding heart or rapid heart rate, sweating, trembling or shaking, feeling of shortness of breath or smothering sensations etc. occur in combination.” D) A person with SAD has “significant anxiety and discomfort about being embarrassed, humiliated, rejected or looked down on in social interactions. People with this disorder will try to avoid the situation or endure it with great anxiety. Common examples are extreme fear of public speaking, meeting new people or eating/drinking in public. The fear or anxiety causes problems with daily functioning and lasts at least six months”.

In the US, 2% of adults are suffering from GAD, 2% suffering from AP, 7% from SAD and 2–3% suffering from PD. National prevalence data indicate that nearly 40 million people in the United States (18%) experience an anxiety disorder in any given year. In addition, according to the World Health Organization (WHO), 1 in 13 globally suffers from anxiety. The WHO reports that anxiety disorders are the most common mental disorders worldwide with specific phobia, major depressive disorder and SAD being the most common anxiety disorders (1). Women are more likely than men to experience anxiety disorder [7].

### Researches related to association between biomarkers and anxiety disorders

Biomarkers are defined as anatomical, biochemical or physiological traits that are specific to certain disorders or syndromes [8]. Biomarkers can be classified as genetic, neuro-imaging, behavioral and biological or blood based. In this study, we have 28 biological biomarkers extracted from blood and urine samples of the subjects.

GAD is still something of an orphan disorder in terms of known biomarkers, as well as in the diagnosis of anxiety disorders [9]. To some extent, this is due to the marked and common overlap of GAD with major depression; also, because the severity of the illness’ impact on the activities of the patients is often overlooked, research funds are limited. Bandelow et al. [8] found that no putative biomarker is sufficient and specific as a diagnostic tool.

### Machine learning & its application in diagnosis of anxiety disorders

**Fig 1:**
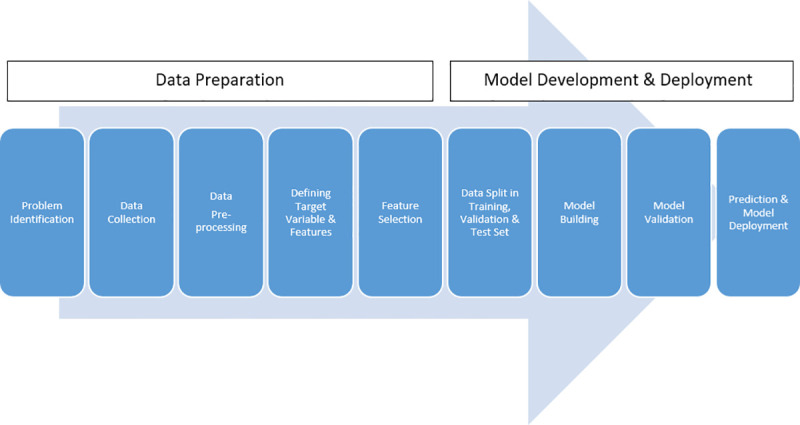
A typical machine learning process. Authors’ own compilation.

Machine Learning is a data-driven approach to solve business problems and a variety of scenarios. Data is the raw material for the machine learning process. A typical machine learning process includes the following steps shown in Fig 1.

The Fig 1 shows a typical process flow of a ML project. ML project starts with problem identification. After defining the problem, the data related to problem identification is collected. Two types of data are collected: data related to the target variable and data related to explanatory variables/ features. The target variable is the data related to the business decisions we want to predict with the ML model. Features are the variables that are used to find an association with target variable through statistical modeling and algorithm. Data needs to be processed so that data gets in the right shape and right format. Features need to be selected so that noise in the data is reduced. After feature selection, model building starts with splitting the dataset into training, validation, and test set. The training set takes 70%-90% observations of the original dataset and it is used for model building. The training set takes 70%-90% of data of the original dataset and it is used for model building. A validation set is a small dataset containing 10%-20% part of the original dataset and it is used to evaluate and validate the ML model. After the satisfactory performance of the ML model, the model is used to predict the target variable. This is the final step of the ML model. If the model performs satisfactorily on the test dataset, the model is deployed to consume unknown data and produce a prediction set of the target variable.

There are two types of ML: Supervised and Unsupervised ML. The process shown in Fig 1 is supervised ML. Supervised ML is used to build models that can predict the target variable based on a set of features. There are two types of supervised ML: Regression-based Supervised ML and Classification based ML. The process mentioned in Fig 1 is a typical workflow of Regression-based Supervised ML. In classification based ML, the features are classified into groups. When features are classified into two groups, it is called Binary Classification and if features are classified into more than two groups, it is called Multi-Class Classification.

In our study, we are using binary classification supervised ML but due to the limitation of data structure and highly imbalanced dataset provided by Lifeline database, we are limiting our machine learning process up to the variable selection step and extraction of variable hierarchy. Anxiety is a mental manifestation and theories in explaining anxiety are still in infancy. The variable importance hierarchy from this study will be an indicative reference list for the researchers to design the experimental studies and prioritize the features.

## Materials and methods

In order to explore the importance of biomarkers’ association with anxiety disorders, we use data from the Dutch Lifelines biobank which collects blood and urine samples, data on brain function as well as self-reports from healthy Dutch citizens. Here, we extracted 28 biomarkers from Lifelines database and bundled the biomarkers into 4 clusters: a) immune system cell counts, b) red blood cell counts, c) biomarkers indicating kidney and liver function d) as well as markers for metabolic disturbances. The dataset extracted from Lifelines databases had more than 28 biomarkers’ data but the majority of them had high proportions of missing values. After data wrangling, the dataset reduced to 28 biomarker variables that were used in the study. So, the choice and number of biomarkers were not arbitrary but the limitation of the dataset provided by the Lifelines database.

The model complexity and model generalizability are the parameters every researcher encounter dealing with ML model-based studies. The model complexity means applying computationally and algorithmically expensive models on the dataset. As we have a small sample of 11081, the model complexities could introduce overfitting in the model, the problem of interpretability, computational inefficiency, and difficulty in comparing the models; we chose simple ML models with default settings so that generalization can be achieved through simple the voting method in computing a list of variable importance of biomarkers with respect to each anxiety disorder. According to No Free Lunch Theorem, there is no one model that works best for all situations, so it is best to test as many as one can [10]. Therefore, we decided to choose the variable importance hierarchy from multiple models.

In ML projects, conventionally, the dataset is divided into two subsets: train and test sets to improve model performance in terms of predictability and the discriminatory power of classification models. In this study, our aim is not to predict the class of anxiety disorder cases and discriminating anxiety cases from healthy cases as the anxiety disorders are fairly complex mental manifestations. As our sample size is small and highly imbalanced, train and test split can create dataset with varying patterns and trends, and comparison of model performance becomes challenging. We wanted to capture the overall pattern and trend of the data without losing an important set of information from the dataset so we performed the variable importance computation based on the complete dataset to extract the variable importance hierarchy. It is theoretically supported by the fact that variable importance extracted from a complete dataset due to the fact that split can produce two subsets with different distributions and variable importance for both subsets may be different. Thus, it will make generalization and comparison variable importance hierarchy extracted from various ML models a challenging task. Complete dataset was used for variable selection and variable importance computation by the researchers [11]. Another research mentioned the method of selecting biomarkers for cancer type detection and extracted the variable importance by applying on the complete dataset [12]. We believe that we need more diverse and more volume of the dataset to build a robust model for the classification of anxiety disorders. In the dataset provided by Lifelines database, there were subjects with multiple anxiety disorders and secondly, we ignored co-morbid cases with other types of mental disorders and physical disorders.

With the majority of studies identified focusing on the detection and diagnosis of mental health conditions, it is evident that there is significant room for applying ML to other areas of psychology and mental health [13]. ML as a subfield of artificial intelligence enables computers to self-learn without being explicitly programmed to do so. When exposed to new data, these computer programs are able to learn, grow, change, and develop research insights by themselves [14].

We applied supervised ML models to create variable importance of biomarkers. However, as each ML technique has both advantages and disadvantages, we aggregate the results by which we create variable importance hierarchy of biomarkers as found in the Lifelines database, according to their ability to predict the four anxieties of interest: A) GAD, B) AP, C) PD and D) SAD.

This research is based on the Lifelines cohort study database. Lifelines is a multi-disciplinary prospective population-based cohort study that uses a unique three-generation design to examine the health and health-related behaviors of persons living in the north of the Netherlands. It employs a broad range of investigative procedures in assessing the biomedical, socio-demographic, behavioral, physical and psychological factors that contribute to the health and diseases among the general population, with a special focus on multi-morbidity and complex genetics. The cohort profile of the Lifelines study is extensively described by Scholtens et al. [15].

Here, we focus on biomarkers that Lifelines has extracted from blood and urine samples and which are part of the Lifelines standard array of diagnostics in profiling a participant’s physical health. We focus on a set of 28 biomarkers from a significant segment of Lifelines’ participants and classified these biomarkers into four clusters: immune system cell counts, red blood cell counts, biomarkers indicating kidney and liver function and those indicating metabolic disturbances. The inclusion versus exclusion criteria are the availability of the biomarkers of interest (from the four clusters of biomarkers) as well as the size of the sample. Concretely, Lifelines does not always collect the biomarkers consistently. For instance, some variables, such as apolipoprotein B100 (ApolipoB100 g/L), free triiodothyronine (Free T3 pmol/L), free thyroxine (Free T4 pmol/L), apolipoprotein A1 (Apolipo g/L), high-sensitivity C-reactive protein (hs-CRP mg/L), and thyroid stimulating hormone (TSH mU/L) had a large number of values missing and thus were removed, so that the overall sample size was not reduced. This resulted in a total study sample of 11,081 participants from the Lifelines baseline data set.

For this research, supervised machine learning is applied. The predictors are a set of biomarker measurements (see Table 1) and the target variable is that of self-reported anxiety disorders with a value of 0 or 1, based on the Mini-International Neuropsychiatric Interview (M.I.N.I.), Dutch Version [16]. The four anxiety disorders were operationalized in the Lifelines database as follows:

- GAD. Respondents were asked: “Have you worried excessively or been anxious about several problems of daily life (problems at work, at home or in your close circle) over the past 6 months?”
- AP. Respondents were asked: “Do you feel anxious and uneasy in places or situations where you might have a panic attack or panic-like symptoms we just spoke about, or where help will not be available or escape might be difficult, like being in a crowd or standing in a queue?”
- PD. Respondents were asked: “Have you, on more than one occasion, had spells or attacks when you suddenly felt anxious, frightened, uncomfortable or uneasy, even in situations where most people would not feel that way? Did the spells surge to a peak within 10 minutes of starting?”
- SAD. Respondents were asked: “In the past month, were you fearful or embarrassed being watched, being the focus of attention, or fearful of being humiliated? This includes things like speaking in public, eating in public or with others, writing while someone watches”.

For all anxiety disorders, the answers were coded “1” for “yes” and “0” for “no” in the Lifelines database. Table 1 describes the variables under study. The total study sample (n = 11,081) consisted of 4,587 male (41.4%) and 6,494 female participants (58.6%) with a mean age of 48.84 years (SD = 11.27).

**Table 1:** Description of predictor variables.

SN	Short Name of Variable	Full Name of Variable and Measurement	Minimum	Maximum	Mean	Standard Deviation
1	AF	Alkaline Phosphatase (U/L)	16.00	356.00	63.18	18.38
2	ALB24	Albumin 24 hrs urine (mg/L)	0.00	2393.00	6.60	45.81
3	ALT	Alanine Aminotransferase (U/L)	2.00	815.00	23.91	20.49
4	AST	Aspartate Aminotransferase ASAT (U/L)	4.00	578.00	24.74	12.25
5	BA	Basophilic Granulocytes (10E9/L)	0.00	0.54	0.03	0.02
6	BALB	Albumin (g/L)	33.00	56.00	44.92	2.37
7	BKR	Creatinine (umol/L)	31.00	474.00	74.18	13.86
8	CA	Calcium (mmol/L)	1.92	2.88	2.28	0.08
9	CHO	Cholesterol (mmol/L)	1.80	10.60	5.14	1.00
10	EO	Eosinophil Granulocytes (10E9/L)	0.00	1.82	0.19	0.12
11	ER	Erythrocytes (10E12/L)	2.67	6.25	4.69	0.38
12	FOS	Phosphate (mmol/L)	0.32	1.57	0.92	0.17
13	GGT	Gamma-GT (U/L)	2.00	1259.00	27.16	28.11
14	GLU	Glucose (mmol/L)	2.70	22.10	5.10	0.86
15	GR	Neutrophil Granulocytes (10E9/L)	0.76	12.63	3.37	1.23
16	HB	Hemoglobin (mmol/L)	4.30	12.20	8.70	0.76
17	HDC	HDL Cholesterol (mmol/L)	0.30	3.70	1.44	0.39
18	HT	Hematocrit (v/v)	0.26	0.55	0.42	0.03
19	K	Potassium (mmol/L)	2.60	5.80	3.92	0.30
20	LDC	LDL Cholesterol (mmol/L)	0.60	8.00	3.31	0.90
21	LY	Lymphocytes (10E9/L)	0.27	5.80	2.02	0.59
22	MO	Monocytes (10E9/L)	0.09	1.59	0.49	0.15
23	NA	Sodium (mmol/L)	126.00	160.00	141.79	1.90
24	TGL	Triglycerides(mmol/L)	0.05	11.85	1.27	0.83
25	TR	Thrombocytes (10E9/L)	41.00	1185.00	252.21	58.04
26	UKR24	Creatinine 24-hrs urine (mmol/L)	1.00	39.10	7.87	3.86
27	UR	Ureum (mmol/L)	1.90	26.70	5.37	1.34
28	UZ	Uric Acid (mmol/L)	0.05	0.65	0.30	0.07

Source: Authors’ own computation from Lifelines Baseline Database. The details about ranges of the biomarkers can be obtained from Lifelines Baseline Database on subscription basis. The researcher cannot share the ranges of biomarker due to restriction distribution condition.

The prevalence of GAD was 20.48% (2271), for AP this was 11.04% (1223), for PD this was 09.20% (1020) and for SAD the prevalence was 05.24% (581) of the total study sample (see the Method section, for more detail).

**Fig 2:**
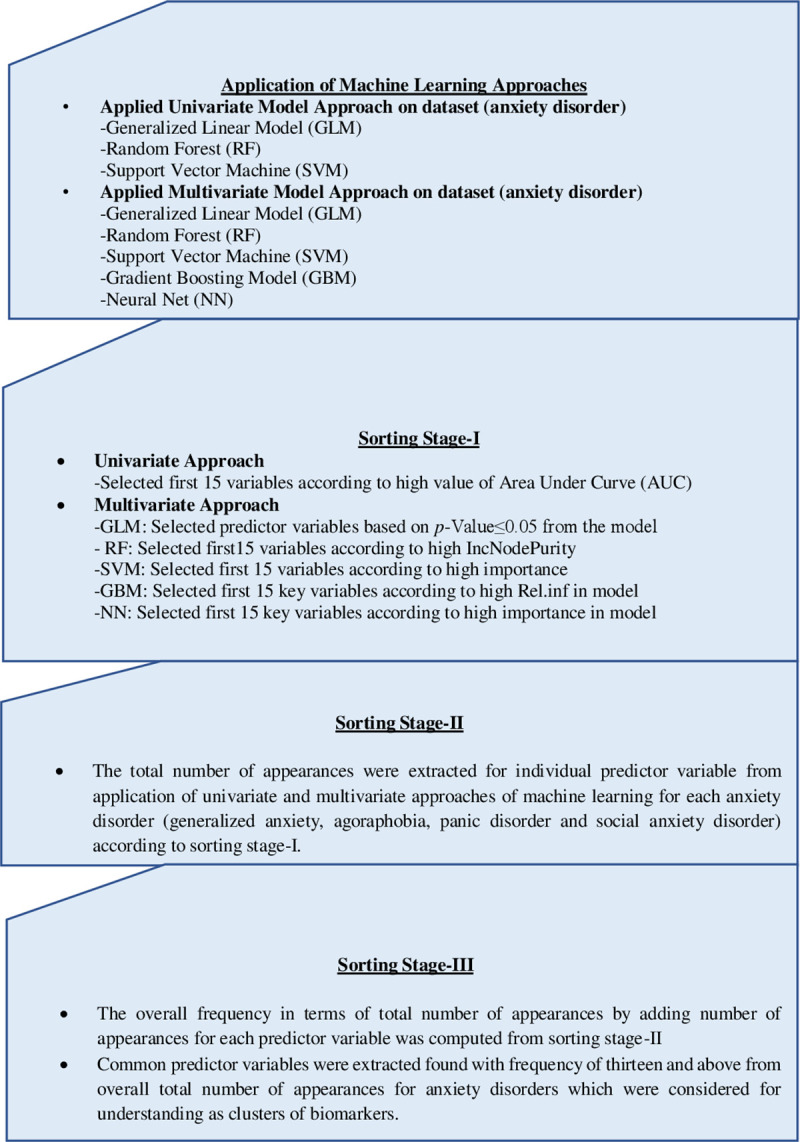
Procedure followed for the study. Authors’ own compilation.

In our model-based approach to exploring the variable importance, we used ML algorithms, a specifically generalized linear model (GLM), random forest (RF), support vector machine (SVM), gradient boosting model (GBM) and neural network (NN). Fig 2 shows the procedure for identifying common predictor variables (biomarker features). For each type of disorder, the predictors were selected on the basis of variable importance tables extracted from the multivariate models approaches.

The Lifelines project provides a cloud-based workspace with software programs for data analysis, such as IBM SPSS and R Programming. We used a simple physical node for our analysis. Given below is the specification of our analysis environment.

- Operating System: Windows 10
- System Type: 64 bits
- Processor: Intel(R) Core (TM) i5 - 8265U
- Processing Speed: 1.60 GHz 1.80 GHz
- RAM: 8 GB
- Development Language: R (version 3.5.0)

## Results

Table 1 in S1 Appendix depicts correlations among different anxiety disorders, using Kendall’s tau-b. For all disorders, we found a positive correlation with a low yet significant value of 0.01. For anxiety disorders, Table 2 in S1 Appendix depicts correlations with body mass index (BMI), age and gender, using Kendall’s tau-b. For GAD, we found a significant positive correlation with body mass index and gender and a significant negative correlation with age. AP showed a significant positive correlation with body mass index and gender. SAD revealed a positive correlation with gender and a negative correlation with age, with 0.01 level of significance. For PD, we found a significant negative correlation with age and significant positive correlation with gender, with 0.01 level of significance.

In the model-based approach, the researcher builds the models of predictor variables with target variable (Anxiety Disorder) and obtains area under curve (AUC) results. The AUC is the area under the receiver operating characteristics (ROC) curve. The value of the AUC curve ranges from 0 to 1. Its value signifies how well the classifier model classifies the two groups: an AUC value that is near “1” or equal to “1” means the classifier model is excellent in classifying the two groups.

We chose AUC measure over ROC measure in spite of the fact that ROC is better diagnostic tool and unbiased towards a particular type of ML models because ROC makes the comparison of multiple ML models challenging [17], we are applying and comparing multiple ML models. AUC score can be compared for binary classifiers and AUC score is most commonly used for comparing classification models on imbalanced datasets [18].

This study considers two main approaches (i.e. the univariate and multivariate approach) to understand the importance of the model-based biomarkers. A univariate approach is used to determine the relationship between the single predictor variable and target variable, and the AUC value is calculated to evaluate variable importance. In a multivariate approach, all predictor variables are entered into the model to predict the target variable. For each target variable, computation was carried out separately.

### Univariate model approach

In the univariate approach, the generalized linear/logistic regression model (GLM)), random forest (RF), and support vector machine (SVM) were used and the AUC with respect to each model was calculated. The AUC scores from each type of model (GLM, RF, and SVM) with respect to each predictor variable are shown in Tables 3–6 in S1 Appendix, for all anxiety disorders. Overall, the AUC scores of random forest models are higher than those from the GLM and SVM models, for all four types of disorders. The highest AUC score was found for neutrophil granulocytes (GR) in RF models and creatinine (BKR) in GLM models, for all anxiety disorders. Creatinine (BKR), hemoglobin (HB), hematocrit (HT) and aspartate aminotransferase (AST) were found with the highest AUC scores in SVM models, for GAD, AP, PD and SAD, respectively.

### Multivariate model approach

In the multivariate model approach, the generalized linear/logistic regression model (GLM), random forest (RF), gradient boosting model (GBM), support vector machine (SVM) and neural network (NN) were used to determine the variable importance for each type of anxiety disorder.

#### Variable importance based on GLM

The logistic regression (GLM) model is used to classify the data set into two groups, the target variable “anxiety disorder” is therefore the binary target variable, as it contains only two values: “0” and “1”. The value “1” means anxiety disorder is diagnosed and “0” means anxiety disorder is not diagnosed. To understand the importance of the variable, the GLM model was applied to both unstandardized and standardized predictors. As shown in Table 1, initially, the model contained 28 predictor variables. Tables 7–10 in S1 Appendix show output of models, for all anxiety disorders. The variables were removed on the basis of minimization according to the Akaike Information Criterion (AIC score) through forward and backward stepwise regression. It was observed that there was no change in AUC measures of both GLM models, for all anxiety disorders. To diagnose the importance of the variable, the standardized estimates of the predictors (biomarker features) were calculated because predictors are measured on various scales. The predictors which were found significant, less than 0.05 level of significance, were taken into consideration for identification of important variables. Tables 7–10 in S1 Appendix show the GLM output based on standardized predictors, and it is evident that low density lipoprotein levels of cholesterol (LDC), calcium (CA), basophilic granulocytes (BA) and phosphate (FOS) have the highest importance for GAD, AP, PD and SAD, respectively.

Creatinine (BKR) was found significant in GLM models, for all anxiety disorders. The calculated AUC value was 0.5865 with a final Akaike Information Criterion (AIC) value of 11091, for GAD, the AUC value from the model for AP was 0.608 with a final AIC value of 7572.5, for PD, the AUC value was 0.6344 with a final AIC value of 6626.1 and, for SAD, the AUC was calculated as 0.591 with a final AIC value of 4520.5.

#### Variable importance based on random forest model

Random forest or random decision forest models constitute an ensemble learning method of classification, regression and other tasks that operates by constructing a multitude of decision trees in model training, and resulting in a classification or mean prediction (regression) of the individual trees [19]. Ensemble learning is based on resampling the data multiple times, building the model on each resample and producing the model with the fewest errors or maximum AUC. The AUC is estimated for binary classification. Due to the multiple applications of bootstrap aggregation or ensembles, the AUC of random forest model prediction of the cases from a set of predictors reached the maximum AUC value of “1”, for all anxiety disorders. In random forest models, variable importance is measured in IncNodePurity. The higher the value of IncNodePurity, the more important the variable. As shown in Table 11 in S1 Appendix, for all anxiety disorder cases, neutrophil granulocytes (GR) was found to have the highest variable importance in predicting, using random forest modeling. Alkaline phosphatase (AF), albumin (ALB24), creatinine (BKR), eosinophil granulocytes (EO), erythrocytes (ER), and lymphocytes (LY) were found common predictor variables (based on high IncNodePurity value) for all anxiety disorders.

#### Variable importance based on the Gradient Boosting Machine

Breiman [20] introduced the Gradient Boosting Machine (GBM). Gradient boosting is a ML technique for regression and classification problems, which produces a prediction model in the form of an ensemble of weak prediction models, typically decision trees. It builds the model in stages, similar to other boosting methods, and generalizes them by allowing optimization of an arbitrary differentiable loss function. The model’s AUC values for GAD, AP, PD and SAD were 0.7714, 0.821, 0.8354 and 0.8737, respectively. The results of GBM for variable importance are shown in Table 12 in S1 Appendix. It is evident that neutrophil granulocytes (GR) have the highest relative influence, compared to any other predictor variable. Creatinine (BKR), eosinophil granulocytes (EO), neutrophil granulocytes (GR), lymphocytes (LY), triglycerides (TGL), thrombocytes (TR), creatinine 24-hours urine (UKR24) and ureum (UR) were commonly found with high relative influence, for all anxiety disorders.

#### Variable importance based on Support Vector Machine

Support Vector Machines are supervised learning models with associated learning algorithms that analyze data used for classification and regression analysis [21]. SVM modeling can also be used for nonlinear models, although only linear SVM was used in this study. The model’s AUC values for GAD, AP, PD and SAD were 0.9228, 0.9515, 0.9596 and 0.8737, respectively. As shown in Table 13 in S1 Appendix, eosinophil granulocytes (EO) have the highest variable importance in predicting cases of GAD and PD, whereas glucose (GLU) and albumin (BALB) have the highest variable importance for AP and SAD, respectively. Basophilic granulocytes (BA), glucose (GLU), triglycerides (TGL), thrombocytes (TR) and sodium were commonly found to have high importance, for all anxiety disorders.

#### Variable importance based on neural network

A neural network is a network or circuit of neurons, or in a modern sense, an artificial neural network, composed of artificial neurons or nodes [22]. Owing to recent developments in computational power, Neural Network (NN) has gained tremendous attention in ML. In this study, the activation function “Softmax” was used. The model AUC value was 0.5892 for GAD, 0.6089 for AP, 0.6407 for PD and 0.5753 for SAD.

As shown in Table 14 in S1 Appendix, the variables with the highest importance were alkaline phosphatase (AF), cholesterol (CHO), low density lipoprotein (LDL Cholesterol) and aspartate aminotransferase (AST), for GAD, AP, PD and SAD, respectively. For all anxiety disorders, alkaline phosphatase (AF) was commonly found with high weight in the neural network model.

## Discussion

The study was aimed to rank the biomarker features in relation to type of anxiety disorders of interest (i.e. GAD, AP, PD and SAD) with the help of ML models and computing variable importance. The variable importance extracted from different models is used to compute the variable importance hierarchy of the biomarker features.

**Fig 3:**
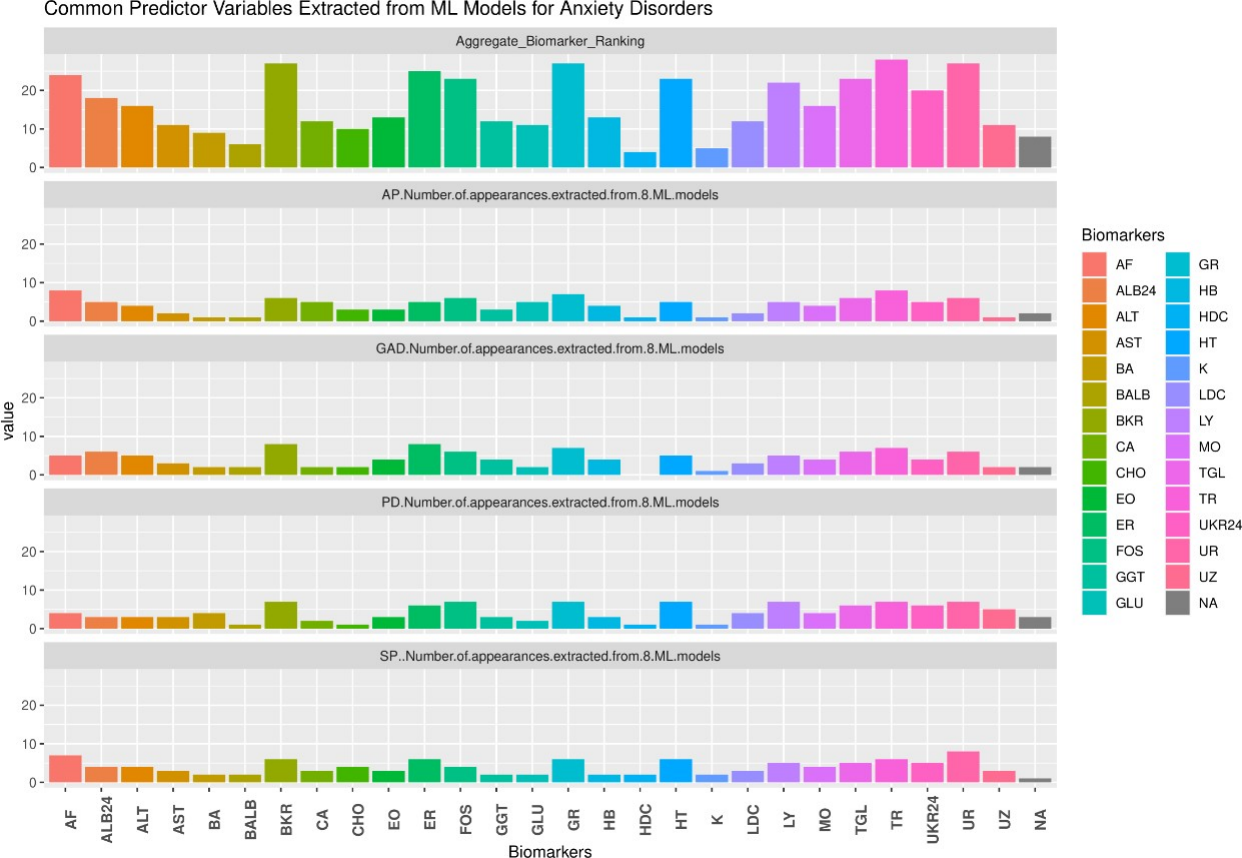
Common predictor variables extracted from ML models for anxiety disorders. Authors’ own computation.

We also explored the associations among the four anxiety disorders. First, the four anxiety disorders of interest have low yet significant correlations (ranging from 0.17 between agoraphobia and generalized anxiety disorder to 0.3 between agoraphobia and panic disorder). This means that the comorbidity of the anxiety disorders is low. Second, based on aggregate machine learning results, we found that the biomarkers within all four clusters of biomarkers were associated with the four anxiety disorders of interest. Third, when aggregating the anxiety disorders of interest into an overall “anxiety disorder”, we found common biomarker features from all four biomarker clusters (See Fig 3).

First, where the immune system cell count is concerned, neutrophil granulocytes (especially important for phagocytosis), monocytes (which enter tissue via blood and transform into macrophages), eosinophil granulocytes (involved in destroying parasites), thrombocytes (or platelets, for cell repair) and finally lymphocytes (which are an aggregate of T, B and natural killer cells) were common predictor variables in that cluster. Second, with respect to the red blood cell count, erythrocytes (a synonym for red blood cells), hematocrit (the ratio between red blood cell volume and total blood volume) and hemoglobin (iron-containing oxygen transporter) were the common predictor variables in that cluster. Third, the common cluster predictors in liver and kidney cells where alkaline phosphatase (an enzyme found in several tissues throughout the body and indicative of liver and kidney malfunctioning), creatinine (found in the blood as a breakdown product due to muscular efforts and its concentration might indicate kidney malfunctioning), ureum (a waste product whose concentration level may indicate both kidney and liver malfunctioning), phosphate (a chemical that contains the mineral phosphorus involved in nerve and muscular function and is filtered and removed by the kidneys, hence it might indicate kidney disease), alanine aminotransferase (an enzyme especially found in the liver), albumin (a globular protein produced by the liver, which might indicate liver malfunctioning) and, finally, creatinine 24-hrs urine (found in urine and might also indicate kidney malfunctioning). Finally, triglycerides (stored in fat cells and indicators of obesity and metabolic syndrome) were the common predictive variable in the metabolic disturbance cluster (Table 2).

**Table 2:** Segmentation of common predictor biomarker variables corresponding to anxiety disorder clusters.

Common Predictor Variables	Identified Clusters
**GR**- Neutrophil Granulocytes (10E9/L)	Immune System Cell Count
**LY**- Lymphocytes (10E9/L)	
**MO**- Monocytes (10E9/L)	
**EO**- Eosinophil Granulocytes (10E9/L)	
**TR**- Thrombocytes (10E9/L)	
**ER**- Erythrocytes (10E12/L)	Red Blood Cell Count(Erythrocytes)
**HT-**Hematocrit(v/v)	
**HB-**Hemoglobin(mmol/L)	
**AF**-Alkaline Phosphatase (U/L)	Biomarkers related to Liver and Kidney related Dysfunction
**BKR**- Creatinine (umol/L)	
**UR**- Ureum (mmol/L)	
**FOS**- Phosphate (mmol/L)	
**ALT**-Alanine Amino Transferase (U/L)	
**ALB24-**Albumin 24 hrs Urine(mg/L)	
**UKR24**- Creatinine 24-hrs urine (mmol/L)	
**TGL**- Triglycerides(mmol/L)	Biomarkers related to Metabolic Disturbances

Source: Authors’ own computation.

Taking a bird’s-eye view, we conjecture that the four clusters of biomarkers that predict anxiety disorders are direct and indirect indicators of the functioning of the immune system, which functions in an M2 or alternative activation state (and produces cytokines like interleukin (IL-10) or interleukin 13 (Il-13)) or an M1 or classical activation state (producing cytokines like interleukin 6 (IL-6) or tumor necrosis factor-α (TNFα)) [23]. We first discuss the immune system functioning in response to peripheral inflammation, then mention briefly how red blood cells affect immune system functioning, and also how liver and kidney malfunctioning and metabolic disturbances affect immune system functioning, and all which affect brain function, followed by the occurrence of anxiety disorders.

First, when encountering threatening stimuli, catecholamines (such as epinephrine or dopamine) are released by the SNS fibers that stimulate bone marrow production and the release of myeloid cells, such as monocytes (which turn into macrophages once they enter certain tissue such as brain tissue). Monocytes and neutrophil granulocytes enter the periphery where they encounter stress-induced damage-associated molecular patterns (DAMPs) and bacteria. This, in turn, activates inflammatory signaling pathways, such as the nuclear factor-kB (NF-kB) and inflammasomes. The activation of inflammasomes leads to the production of mature cytokines (such as interleukin 18 (IL-18)) and the activation of NF-kB which stimulates the release of pro-inflammatory cytokines, such as TNF and IL-6. Both can access the brain through humoral (e.g., brain parenchyma in areas where there is a lack of intact blood–brain barrier, such as in the circumventricular organ) and neural routes (e.g., vagus nerve). From here, the glia (oligodendrocytes, astrocytes, ependymal cells and microglia) is activated and subsequently turn glia activation from an M2 state (anti-inflammatory) to an M1 state, the latter of which is called the inflammatory state and, thus, also evokes the release of cytokines (IL-6 or TNFα). Once in an M1 state, the glia (or macrophages) release chemokines, which attract monocytes to the brain via the cellular route [freely adopted from 24].

M1 glia (or macrophages) can perpetuate CNS inflammatory responses which influence neurotransmitter systems, such as the serotonin system that affects anxiety-related network functioning [25]. For instance, once affected by cytokines such as TNFα or interleukin 1 beta (IL-1B), glia (e.g., microglia) reduce the availability of monoamines such as serotonin, dopamine and noradrenaline, as they increase the presynaptic reuptake pumps for these neurotransmitters, all of which are part of the anxiety network. Second, activated glia (e.g., astrocytes) deregulate *N*-methyl-D-aspartate receptors (NMDAR) and also reduce glutamate reuptake which, in turn, leads to decreased BDNF in the hippocampus and this affects learning new ways of coping with stress.

The literature already conceives the role of erythrocytes as directly participating in the immune complex reaction (bacteria, complement and antibody). These erythrocytes play a role in clearing pathogens by macrophages and also produce cytokines or specific signaling molecules [26, 27]. In addition, pathogens that are destroyed become a target for the erythrocytes for transport throughout the organism [28].

In addition, liver malfunction and kidney function also greatly affect the immune system. The kidneys and the brain share similar hemodynamics in their vascular supply and kidney impairment comes with M1 activity due to pro-inflammatory cytokines (e.g., interleukin 2 (IL-2) and interleukin 10 (IL-10) as well as pro-inflammatory chemokines (e.g., CXCL1)), which reach the glia in the brain and turn the M2 state of e.g., microglia and astrocytes into an M1 state, instigating the described effects on brain functioning, such as mono amine metabolism and this, in turn, amplifies the anxiety system [freely adopted from 29].

Liver malfunction comes with specific immune-mediated pathways that affect the microglia balance shifting from M2 to M1, as they respond to the pro-inflammatory cytokines tumor necrosis factor-α (TNFα), IL-1β and IL-6 [30, 31]. For instance, vagal afferents project to the dorsal vagal complex, which includes tractus solitarius or dorsal motor areas and, from there, they reach certain regions of the brain, such as the paraventricular nucleus of the hypothalamus. In addition, the mentioned cytokines also gain access to the brain via permeable blood-brain barriers and also interact with their receptors on the endothelial cells, thus creating signaling pathways. Once the microglia in the brain are hyper-activated (switch from M2 to M1), they attract more monocytes via monocyte chemoattractant, thus generating inflammation in the brain (M1 macrophage activation) [24].

Finally, metabolic disturbances predict anxiety and, here, triglycerides are the significant biomarker features. This marker, amongst others, is indicative of metabolic syndrome, which is known as a cluster of conditions occurring together, increasing people’s risk of heart disease, stroke and type 2 diabetes. These conditions include increased excess body fat around the waist, and abnormal triglyceride levels [32]. Higher triglyceride molecules are related to obesity and obesity that leads to chronic inflammation [33]. People with a low body mass index (BMI) have adipose tissue that expresses M2 macrophages (stimulated by, e.g., cytokine interleukin 4 (Il-4)) whereas obese people recruit M1 macrophages (evoked by e.g. cytokine interferon-γ (IFNγ) and obese people shift the M1/M2 balance in favor of M1 [34]. These pro-inflammatory cytokines (such as IFNγ or TNFα) then reach the brain via the humoral, neural and cellular routes, where they cause the already mentioned shift in balance from M2 to M1 activation in microglia (or macrophages), thus, causing anxiety [33].

## Conclusions

We found that the “common biomarker predictors” were found with each type of anxiety disorder selected in the study. Having described the results, we discuss how our findings might add to our understanding of these anxiety disorders.

The study was aimed to rank the biomarker features in relation to type of anxiety disorders of interest (i.e. GAD, AP, PD and SAD) with the help of ML models and computing variable importance.

We also explored the associations among the four anxiety disorders. First, the four anxiety disorders of interest have low yet significant correlations (ranging from 0.17 between AP and GAD to 0.3 between AP and PD). This means that the comorbidity of the anxiety disorders is low. Second, based on aggregate ML results, we found that the biomarkers within all four clusters of biomarkers were commonly associated with the four anxiety disorders of interest (See Fig 3). Third, when aggregating the anxiety disorders of interest into an overall “anxiety disorder”, we found common biomarker features from all four biomarker clusters (See Table 2).

Future research work on anxiety disorders should be based on building a robust classification model with larger volume datasets with more features than used in this study. The study was only focused on the dataset related to Dutch citizens which limits the generalization of the study to other countries. Anxiety disorder is affecting a very large section of the population in the world hence the model development for anxiety diagnosis and classification should be taken at war footing. In this study, we could focus only on the variable importance hierarchy of biomarkers in diagnosing the anxiety disorder, the future studies can be focused on external validation of the findings through clinical based research as anxiety disorders are complex disorders and merely 28 biomarkers cannot build reliable models. In future studies, more features related to genes, lifestyle habits, environmental aspects, social-cultural aspects, etc. should be included to build robust diagnostic models for anxiety disorders.

## Supporting information

S1 Appendix **Table 1–Table 14.** Authors’ own computation.
(DOCX)

S2 Appendix Machine learning models: Default settings. (DOCX)
