# Supplementary material for: Understanding importance of clinical biomarkers for diagnosis of anxiety disorders using machine learning models
Source: PLoS One. 2021 May 10;16(5):e0251365. doi: 10.1371/journal.pone.0251365 (PMC8109802; doi:10.1371/journal.pone.0251365)
Supplement: S1 Appendix — Table 1–Table 14. Authors’ own computation. (DOCX) [file pone.0251365.s001.docx]

**S1 Appendix**

**Table 1. Correlation among Anxiety Disorders.**

| **Anxiety Disorders** | **Kendall Tau-b Correlation** | **Generalized Anxiety Disorder**  **(GAD)** | **Agoraphobia (AP)** | **Social Anxiety Disorder**  **(SAD)** | **Panic Disorder**  **(PD)** |
| --- | --- | --- | --- | --- | --- |
| **Generalized Anxiety Disorder (GAD)** | Correlation Coefficient | 1 | .166^**^ | .213^**^ | .216^**^ |
|  | Sig. (2-tailed) |  | 0.000 | 0.000 | 0.000 |
|  | N | 11081 | 11081 | 11081 | 11081 |
| **Agoraphobia (AP)** | Correlation Coefficient | .166^**^ | 1 | .194^**^ | .300^**^ |
|  | Sig. (2-tailed) | 0.000 |  | 0.000 | 0.000 |
|  | N | 11081 | 11081 | 11081 | 11081 |
| **Social Anxiety Disorder (SAD)** | Correlation Coefficient | .213^**^ | .194^**^ | 1 | .170^**^ |
|  | Sig. (2-tailed) | 0.000 | 0.000 |  | 0.000 |
|  | N | 11081 | 11081 | 11081 | 11081 |
| **Panic Disorder (PD)** | Correlation Coefficient | .216^**^ | .300^**^ | .170^**^ | 1 |
|  | Sig. (2-tailed) | 0.000 | 0.000 | 0.000 |  |
|  | N | 11081 | 11081 | 11081 | 11081 |

** Correlation is significant at the 0.01 level (2-tailed).

Source: Authors’ own computation.

**Table 2. Correlation of Anxiety Disorders with BMI, Age and Gender.**

| **Anxiety Disorders** | **Kendall Tau-b Correlation** | **BMI (kg/M^2)** | **Age** | **Gender** |
| --- | --- | --- | --- | --- |
| **Generalized Anxiety Disorder (GAD)** | Correlation Coefficient | .024^*^ | -.073^**^ | .079^**^ |
|  | Sig. (2-tailed) | 0.012 | 0.000 | 0.000 |
|  | N | 11077 | 11081 | 11081 |
| **Agoraphobia (AP)** | Correlation Coefficient | .034^**^ | 0.008 | .098^**^ |
|  | Sig. (2-tailed) | 0.000 | 0.403 | 0.000 |
|  | N | 11077 | 11081 | 11081 |
| **Social Anxiety Disorder (SAD)** | Correlation Coefficient | 0.015 | -.058^**^ | .047^**^ |
|  | Sig. (2-tailed) | 0.122 | 0.000 | 0.000 |
|  | N | 11077 | 11081 | 11081 |
| **Panic Disorder (PD)** | Correlation Coefficient | -0.007 | -.073^**^ | .095^**^ |
|  | Sig. (2-tailed) | 0.490 | 0.000 | 0.000 |
|  | N | 11077 | 11081 | 11081 |

** Correlation is significant at the 0.01 level (2-tailed).

* Correlation is significant at the 0.05 level (2-tailed).

Source: Authors’ own computation.

**Table 3. AUC Comparisons of Univariate Models for Generalized Anxiety Disorder (GAD).**

| **S.N.** | **Predictors** | **AUC_GLM** | **Predictors** | **AUC_RF** | **Predictors** | **AUC_SVM** |
| --- | --- | --- | --- | --- | --- | --- |
| 1 | BKR | 0.5539 | GR | 0.6599 | BKR | 0.5322 |
| 2 | UR | 0.5459 | TGL | 0.6257 | GR | 0.5299 |
| 3 | TR | 0.5444 | TR | 0.6178 | HB | 0.5246 |
| 4 | GR | 0.5387 | LY | 0.6144 | AST | 0.5234 |
| 5 | LY | 0.5352 | UKR24 | 0.5970 | ALT | 0.5218 |
| 6 | ER | 0.5341 | HT | 0.5957 | TR | 0.5189 |
| 7 | UZ | 0.5302 | ER | 0.5946 | CA | 0.5188 |
| 8 | HB | 0.5293 | ALB24 | 0.5799 | ER | 0.5138 |
| 9 | AST | 0.5269 | BKR | 0.5758 | ALB24 | 0.5125 |
| 10 | HT | 0.5261 | UR | 0.5694 | AF | 0.5122 |
| 11 | ALT | 0.5250 | ALT | 0.5691 | LDC | 0.5119 |
| 12 | ALB24 | 0.5181 | GGT | 0.5682 | UZ | 0.5115 |
| 13 | FOS | 0.5180 | FOS | 0.5653 | TGL | 0.5106 |
| 14 | NA | 0.5172 | MO | 0.5597 | FOS | 0.5098 |
| 15 | BALB | 0.5167 | AF | 0.5595 | HT | 0.5095 |
| 16 | CA | 0.5158 | CA | 0.5526 | CHO | 0.5093 |
| 17 | BA | 0.5148 | EO | 0.5506 | BALB | 0.5092 |
| 18 | TGL | 0.5147 | HB | 0.5503 | BA | 0.5088 |
| 19 | HDC | 0.5130 | LDC | 0.5502 | NA | 0.5084 |
| 20 | AF | 0.5102 | UZ | 0.5482 | K | 0.5079 |
| 21 | CHO | 0.5079 | AST | 0.5478 | UKR24 | 0.5062 |
| 22 | GGT | 0.5070 | CHO | 0.5436 | GGT | 0.5031 |
| 23 | MO | 0.5063 | GLU | 0.5399 | GLU | 0.5020 |
| 24 | GLU | 0.5054 | HDC | 0.5296 | MO | 0.5005 |
| 25 | LDC | 0.5053 | NA | 0.5254 | UR | 0.4996 |
| 26 | K | 0.5043 | BALB | 0.5244 | LY | 0.4967 |
| 27 | UKR24 | 0.5012 | K | 0.5229 | EO | 0.4966 |
| 28 | EO | 0.4945 | BA | 0.5203 | HDC | 0.4910 |

Source: Authors’ own computation.

**Table 4. AUC Comparisons of Univariate Models for Agoraphobia (AP).**

| **S.N.** | **Predictors** | **AUC_GLM** | **Predictors** | **AUC_RF** | **Predictors** | **AUC_SVM** |
| --- | --- | --- | --- | --- | --- | --- |
| 1 | BKR | 0.5804 | GR | 0.7030 | HB | 0.5355 |
| 2 | ER | 0.5508 | TGL | 0.6613 | UKR24 | 0.5312 |
| 3 | TR | 0.5481 | LY | 0.6513 | UR | 0.5230 |
| 4 | HB | 0.5436 | TR | 0.6406 | TR | 0.5202 |
| 5 | UKR24 | 0.5424 | ER | 0.6206 | GR | 0.5157 |
| 6 | HT | 0.5401 | UKR24 | 0.6199 | LDC | 0.5144 |
| 7 | UZ | 0.5361 | HT | 0.6172 | FOS | 0.5144 |
| 8 | UR | 0.5332 | BKR | 0.6033 | BKR | 0.5120 |
| 9 | GR | 0.5327 | ALB24 | 0.5950 | TGL | 0.5117 |
| 10 | FOS | 0.5315 | GGT | 0.5882 | AF | 0.5102 |
| 11 | LY | 0.5297 | AF | 0.5835 | GLU | 0.5097 |
| 12 | AF | 0.5274 | FOS | 0.5806 | CA | 0.5081 |
| 13 | BALB | 0.5268 | UR | 0.5799 | K | 0.5067 |
| 14 | AST | 0.5242 | MO | 0.5782 | ALB24 | 0.5067 |
| 15 | ALT | 0.5241 | ALT | 0.5744 | HT | 0.5062 |
| 16 | TGL | 0.5223 | HB | 0.5720 | ALT | 0.5038 |
| 17 | GLU | 0.5175 | CHO | 0.5668 | EO | 0.5036 |
| 18 | MO | 0.5163 | EO | 0.5662 | LY | 0.5023 |
| 19 | CHO | 0.5127 | UZ | 0.5658 | MO | 0.5010 |
| 20 | BA | 0.5110 | CA | 0.5652 | GGT | 0.5007 |
| 21 | NA | 0.5104 | AST | 0.5562 | NA | 0.4990 |
| 22 | ALB24 | 0.5094 | GLU | 0.5557 | ER | 0.4965 |
| 23 | CA | 0.5087 | LDC | 0.5552 | UZ | 0.4963 |
| 24 | HDC | 0.5073 | HDC | 0.5440 | BALB | 0.4959 |
| 25 | K | 0.5022 | BALB | 0.5396 | HDC | 0.4927 |
| 26 | GGT | 0.5013 | NA | 0.5368 | CHO | 0.4926 |
| 27 | EO | 0.4988 | K | 0.5364 | AST | 0.4873 |
| 28 | LDC | 0.4983 | BA | 0.5187 | BA | 0.4856 |

Source: Authors’ own computation.

**Table 5. AUC Comparisons of Univariate Models for Panic Disorder (PD).**

| **S.N.** | **Predictors** | **AUC_GLM** | **Predictors** | **AUC_RF** | **Predictors** | **AUC_SVM** |
| --- | --- | --- | --- | --- | --- | --- |
| 1 | BKR | 0.5922 | GR | 0.7262 | HT | 0.5432 |
| 2 | UR | 0.5810 | TR | 0.6660 | HB | 0.5335 |
| 3 | UZ | 0.5706 | LY | 0.6648 | AST | 0.5325 |
| 4 | ER | 0.5599 | TGL | 0.6528 | UZ | 0.5318 |
| 5 | UKR24 | 0.5594 | ER | 0.6482 | HDC | 0.5297 |
| 6 | FOS | 0.5573 | HT | 0.6230 | UKR24 | 0.5247 |
| 7 | TR | 0.5541 | UKR24 | 0.6179 | GGT | 0.5244 |
| 8 | HB | 0.5510 | BKR | 0.6131 | TR | 0.5228 |
| 9 | HT | 0.5508 | UR | 0.6107 | FOS | 0.5226 |
| 10 | GR | 0.5397 | GGT | 0.5978 | GR | 0.5189 |
| 11 | AST | 0.5386 | FOS | 0.5962 | BKR | 0.5179 |
| 12 | NA | 0.5365 | ALB24 | 0.5922 | UR | 0.5163 |
| 13 | LY | 0.5315 | MO | 0.5867 | MO | 0.5159 |
| 14 | ALT | 0.5269 | UZ | 0.5866 | AF | 0.5103 |
| 15 | BA | 0.5231 | ALT | 0.5845 | GLU | 0.5096 |
| 16 | GLU | 0.5209 | HB | 0.5823 | BALB | 0.5092 |
| 17 | TGL | 0.5188 | AF | 0.5809 | ALB24 | 0.5078 |
| 18 | GGT | 0.5166 | AST | 0.5729 | EO | 0.5068 |
| 19 | K | 0.5147 | EO | 0.5691 | LDC | 0.5054 |
| 20 | BALB | 0.5141 | CHO | 0.5644 | TGL | 0.5045 |
| 21 | CA | 0.5109 | LDC | 0.5600 | BA | 0.5034 |
| 22 | HDC | 0.5086 | GLU | 0.5576 | CA | 0.5026 |
| 23 | ALB24 | 0.5076 | CA | 0.5570 | LY | 0.5014 |
| 24 | EO | 0.5063 | HDC | 0.5489 | CHO | 0.5002 |
| 25 | CHO | 0.5054 | K | 0.5403 | NA | 0.4975 |
| 26 | AF | 0.5031 | NA | 0.5395 | ER | 0.4966 |
| 27 | MO | 0.5018 | BA | 0.5366 | K | 0.4949 |
| 28 | LDC | 0.4979 | BALB | 0.5272 | ALT | 0.4902 |

Source: Authors’ own computation.

**Table 6. AUC Comparisons of Univariate Models for Social Anxiety Disorder (SAD).**

| **S.N.** | **Predictors** | **AUC_GLM** | **Predictors** | **AUC_RF** | **Predictors** | **AUC_SVM** |
| --- | --- | --- | --- | --- | --- | --- |
| 1 | BKR | 0.5615 | GR | 0.7794 | AST | 0.5345 |
| 2 | UR | 0.5543 | TGL | 0.7086 | LDC | 0.5316 |
| 3 | UZ | 0.5455 | LY | 0.7060 | ALB24 | 0.5283 |
| 4 | AST | 0.5408 | TR | 0.6955 | ALT | 0.5271 |
| 5 | GR | 0.5394 | ER | 0.6680 | UZ | 0.5238 |
| 6 | HT | 0.5381 | HT | 0.6612 | TR | 0.5237 |
| 7 | ER | 0.5376 | UKR24 | 0.6565 | GGT | 0.5225 |
| 8 | HB | 0.5361 | ALB24 | 0.6372 | UR | 0.5203 |
| 9 | FOS | 0.5288 | AF | 0.6144 | GR | 0.5187 |
| 10 | ALT | 0.5274 | GGT | 0.6116 | CHO | 0.5186 |
| 11 | TR | 0.5267 | BKR | 0.6072 | ER | 0.5152 |
| 12 | CHO | 0.5250 | FOS | 0.6032 | AF | 0.5148 |
| 13 | LDC | 0.5243 | MO | 0.6017 | K | 0.5104 |
| 14 | UKR24 | 0.5228 | UR | 0.5956 | BALB | 0.5104 |
| 15 | AF | 0.5222 | HB | 0.5917 | LY | 0.5081 |
| 16 | CA | 0.5220 | ALT | 0.5913 | TGL | 0.5055 |
| 17 | GGT | 0.5195 | EO | 0.5861 | BA | 0.5055 |
| 18 | NA | 0.5192 | LDC | 0.5848 | UKR24 | 0.5053 |
| 19 | MO | 0.5166 | CA | 0.5821 | HB | 0.5048 |
| 20 | LY | 0.5164 | CHO | 0.5802 | CA | 0.5047 |
| 21 | GLU | 0.5117 | AST | 0.5775 | GLU | 0.5046 |
| 22 | K | 0.5095 | UZ | 0.5764 | MO | 0.5046 |
| 23 | TGL | 0.5081 | GLU | 0.5761 | HDC | 0.5035 |
| 24 | EO | 0.5074 | K | 0.5561 | NA | 0.5013 |
| 25 | HDC | 0.5053 | NA | 0.5521 | HT | 0.4986 |
| 26 | BA | 0.4979 | HDC | 0.5504 | BKR | 0.4961 |
| 27 | BALB | 0.4979 | BALB | 0.5350 | FOS | 0.4960 |
| 28 | ALB24 | 0.4854 | BA | 0.5228 | EO | 0.4945 |

Source: Authors’ own computation.

**Table 7. GLM Model Output for Standardized Predictors for Generalized Anxiety Disorder (GAD).**

| **Predictors** | **Estimate** | **Std. Error** | **Z Value** | **P-Value** | **Significance** |
| --- | --- | --- | --- | --- | --- |
| Intercept | 0.001582 | 0.4865 | 0.003 | 0.9974 |  |
| BKR | -22.3300 | 5.4220 | -4.118 | 0.0000 | *** |
| LY | 406.3000 | 107.8000 | 3.768 | 0.0002 | *** |
| GR | 203.3000 | 55.2700 | 3.678 | 0.0002 | *** |
| ER | -936.7000 | 259.6000 | -3.607 | 0.0003 | *** |
| CHO | -551.2000 | 158.0000 | -3.487 | 0.0005 | *** |
| MO | -1561.0000 | 473.3000 | -3.299 | 0.0009 | *** |
| LDC | 551.9000 | 169.7000 | 3.253 | 0.0011 | ** |
| UR | -159.8000 | 49.8800 | -3.205 | 0.0014 | ** |
| TGL | 248.8000 | 78.6300 | 3.164 | 0.0016 | ** |
| TR | 2.6230 | 1.0550 | 2.487 | 0.0129 | * |
| HT | 5953.0000 | 3343.0000 | 1.781 | 0.0750 | . |
| FOS | 603.2000 | 371.8000 | 1.623 | 0.1047 |  |
| AF | 5.1490 | 3.2690 | 1.575 | 0.1152 |  |
| GLU | -113.7000 | 73.1100 | -1.555 | 0.1200 |  |
| GGT | 2.9770 | 2.0510 | 1.451 | 0.1467 |  |

**Signif. codes: 0 '***' 0.001 '**' 0.01 '*' 0.05 '.' 0.1 ' ' 1**

Source: Authors’ own computation.

**Table 8. GLM Model Output from Standardized Predictors for Agoraphobia (AP).**

| **Predictors** | **Estimate** | **Std. Error** | **Z Value** | **P-Value** | **Significance** |
| --- | --- | --- | --- | --- | --- |
| Intercept | -2.2978 | 0.9482 | -2.423 | 0.015373 | * |
| BKR | -47.1738 | 6.6559 | -7.087 | 0.0000 | *** |
| HDC | -849.4855 | 251.5477 | -3.377 | 0.0007 | *** |
| ER | -1015.6719 | 335.5614 | -3.027 | 0.0025 | ** |
| CHO | 658.4097 | 222.1196 | 2.964 | 0.0030 | ** |
| AF | 11.4066 | 3.9498 | 2.888 | 0.0039 | ** |
| LDC | -622.2282 | 244.0979 | -2.549 | 0.0108 | * |
| CA | 2501.5544 | 1004.6715 | 2.490 | 0.0128 | * |
| TR | 3.0133 | 1.2438 | 2.423 | 0.0154 | * |
| GLU | 164.4038 | 77.4446 | 2.123 | 0.0338 | * |
| ALT | -11.4066 | 5.5293 | -2.063 | 0.0391 | * |
| BALB | -64.6517 | 36.1964 | -1.786 | 0.0741 | . |
| HT | 7247.8488 | 4203.2005 | 1.724 | 0.0846 |  |
| FOS | 704.0810 | 473.0141 | 1.488 | 0.1366 |  |

**Signif. codes: 0 '***' 0.001 '**' 0.01 '*' 0.05 '.' 0.1 ' ' 1**

Source: Authors’ own computation.

**Table 9. GLM Model Output from Standardized Predictors for Panic Disorder (PD).**

| **Predictors** | **Estimate** | **Std. Error** | **Z Value** | **P-Value** | **Significance** |
| --- | --- | --- | --- | --- | --- |
| Intercept | 4.443 | 2.506 | 1.773 | 0.07629 | . |
| UR | -367.5560 | 73.644 | -4.991 | 0.0000 | *** |
| FOS | 2528.4890 | 518.496 | 4.877 | 0.0000 | *** |
| TGL | 447.3670 | 106.429 | 4.203 | 0.0000 | *** |
| BKR | -26.1980 | 8.135 | -3.220 | 0.0013 | ** |
| LDC | 622.1220 | 236.152 | 2.634 | 0.0084 | ** |
| GR | 195.2190 | 74.483 | 2.621 | 0.0088 | ** |
| UZ | -3800.9930 | 1454.777 | -2.613 | 0.0090 | ** |
| LY | 337.0670 | 147.106 | 2.291 | 0.0220 | * |
| BA | 8297.2230 | 3655.315 | 2.270 | 0.0232 | * |
| ER | -563.8380 | 249.341 | -2.261 | 0.0237 | * |
| NA | -82.4800 | 42.643 | -1.934 | 0.0531 | . |
| CHO | -415.1840 | 220.260 | -1.885 | 0.0594 | . |
| MO | -1196.0960 | 660.255 | -1.812 | 0.0701 | . |
| GLU | -157.5260 | 106.813 | -1.475 | 0.1403 |  |

**Signif. codes: 0 '***' 0.001 '**' 0.01 '*' 0.05 '.' 0.1 ' ' 1**

Source: Authors’ own computation.

**Table 10. GLM Model Output from Standardized Predictors for Social Anxiety Disorder (SAD).**

| **Predictors** | **Estimate** | **Std. Error** | **Z Value** | **P-Value** | **Significance** |
| --- | --- | --- | --- | --- | --- |
| Intercept | -1.572 | 1.263 | 1.245 | 0.21299 |  |
| GR | 203.4910 | 78.888 | 2.579 | 0.0099 | ** |
| BKR | -25.2170 | 10.139 | -2.487 | 0.0129 | * |
| UR | -214.5620 | 91.672 | -2.341 | 0.0193 | * |
| TGL | 255.1270 | 117.381 | 2.173 | 0.0297 | * |
| FOS | 1320.3610 | 647.689 | 2.039 | 0.0415 | * |
| BALB | 92.9190 | 49.569 | 1.875 | 0.0609 | . |
| CA | -2564.5900 | 1440.740 | -1.780 | 0.0751 | . |
| UZ | -2689.6880 | 1790.353 | -1.502 | 0.1330 |  |

**Signif. codes: 0 '***' 0.001 '**' 0.01 '*' 0.05 '.' 0.1 ' ' 1**

Source: Authors’ own computation.

**Table 11. Random Forest Model (RF) Output for Variable Importance.**

| **Anxiety Disorders** | **Generalized Anxiety Disorder**  **(GAD)** | | **Agoraphobia**  **(AP)** | | **Panic Disorder**  **(PD)** | | **Social Anxiety Disorder**  **(SAD)** | |
| --- | --- | --- | --- | --- | --- | --- | --- | --- |
| **SN** | **Variable** | **IncNodePurity** | **Variable** | **IncNodePurity** | **Variable** | **IncNodePurity** | **Variable** | **IncNodePurity** |
| 1 | GR | 78.5370 | LY | 44.7583 | GR | 39.2190 | TGL | 23.4613 |
| 2 | LY | 78.0756 | UKR24 | 43.8433 | LY | 38.6641 | TR | 21.8048 |
| 3 | TR | 76.8921 | GR | 43.7518 | UKR24 | 37.2922 | GR | 21.7744 |
| 4 | UKR24 | 75.6114 | TGL | 43.0539 | TGL | 36.4874 | AF | 21.4050 |
| 5 | TGL | 74.4909 | TR | 42.6486 | FOS | 35.2021 | LY | 20.9354 |
| 6 | AF | 70.6625 | AF | 41.9897 | TR | 34.8253 | ER | 20.8724 |
| 7 | ER | 69.2150 | FOS | 41.4350 | EO | 33.7536 | UKR24 | 20.2980 |
| 8 | FOS | 67.2865 | ER | 40.3206 | MO | 33.7180 | EO | 19.6528 |
| 9 | ALB24 | 66.5148 | MO | 40.2322 | BKR | 33.5243 | UR | 19.0381 |
| 10 | UR | 65.7708 | UR | 39.7832 | ER | 33.3455 | HT | 18.4978 |
| 11 | EO | 65.0256 | BKR | 39.5054 | UR | 33.3336 | BKR | 18.3265 |
| 12 | BKR | 64.4952 | CA | 37.9538 | AF | 33.2729 | MO | 18.2942 |
| 13 | MO | 64.1318 | ALB24 | 37.3572 | ALB24 | 33.1794 | CA | 18.1930 |
| 14 | HT | 61.0719 | EO | 37.1465 | CA | 30.5691 | FOS | 17.7097 |
| 15 | GGT | 60.7004 | HT | 36.8400 | HT | 29.7534 | ALB24 | 17.5491 |
| 16 | ALT | 57.5936 | ALT | 32.9666 | GGT | 28.8083 | CHO | 16.7904 |
| 17 | CA | 57.5872 | UZ | 32.8406 | UZ | 28.8070 | ALT | 16.7315 |
| 18 | UZ | 57.3724 | GGT | 32.7086 | ALT | 28.2967 | GLU | 16.4207 |
| 19 | GLU | 55.4028 | CHO | 32.2254 | LDC | 27.8101 | GGT | 16.1624 |
| 20 | CHO | 54.0238 | GLU | 31.8363 | CHO | 27.3962 | LDC | 15.7547 |
| 21 | LDC | 52.5734 | K | 31.1276 | AST | 26.5930 | AST | 15.4684 |
| 22 | AST | 50.4851 | LDC | 31.0305 | GLU | 25.6807 | K | 14.5182 |
| 23 | K | 47.0782 | HB | 27.9942 | HB | 23.1685 | UZ | 14.2294 |
| 24 | HB | 46.7536 | AST | 27.1728 | HDC | 22.8540 | HB | 13.5296 |
| 25 | HDC | 45.6346 | HDC | 26.7527 | K | 22.8033 | HDC | 13.1685 |
| 26 | BALB | 39.2469 | NA | 23.5197 | NA | 19.7392 | NA | 12.8610 |
| 27 | NA | 36.0213 | BALB | 22.7510 | BALB | 19.7333 | BALB | 11.9467 |
| 28 | BA | 33.7286 | BA | 21.3631 | BA | 18.8546 | BA | 9.4996 |

Source: Authors’ own computation.

**Table 12. Gradient Boosting Model (GBM) Output for Variable Importance.**

| **A.D.** | **Generalized Anxiety Disorder**  **(GAD)** | | **Agoraphobia**  **(AP)** | | **Panic Disorder**  **(PD)** | | **Social Anxiety Disorder**  **(SAD)** | |
| --- | --- | --- | --- | --- | --- | --- | --- | --- |
| **SN** | **Variable** | **Rel.inf** | **Variable** | **Rel.inf** | **Variable** | **Rel.inf** | **Variable** | **Rel.inf** |
| 1 | GR | 6.8998 | LY | 7.3810 | LY | 7.3944 | EO | 15.1682 |
| 2 | TGL | 6.1466 | UKR24 | 5.8308 | GR | 6.3458 | TGL | 7.2280 |
| 3 | UKR24 | 5.6045 | GR | 5.6225 | EO | 5.8256 | UR | 6.6330 |
| 4 | LY | 5.5855 | AF | 5.5263 | UR | 5.7030 | ER | 5.7657 |
| 5 | AF | 5.5046 | UR | 5.4953 | TGL | 5.2597 | BKR | 5.4926 |
| 6 | TR | 5.4412 | BKR | 5.0337 | UKR24 | 5.2333 | GR | 4.8728 |
| 7 | ER | 5.3999 | MO | 4.7110 | FOS | 4.9683 | CHO | 4.7694 |
| 8 | HT | 4.4565 | TGL | 4.5526 | BKR | 4.5585 | LY | 4.3593 |
| 9 | ALT | 4.3314 | FOS | 4.5151 | TR | 4.5137 | TR | 3.7835 |
| 10 | FOS | 3.9804 | TR | 4.4126 | UZ | 4.3989 | CA | 3.7642 |
| 11 | EO | 3.9568 | EO | 3.8261 | MO | 4.2428 | AF | 3.7340 |
| 12 | BKR | 3.9100 | ALT | 3.6754 | ER | 4.2167 | HT | 3.3422 |
| 13 | UR | 3.7604 | GGT | 3.5459 | LDC | 3.4957 | ALT | 3.0908 |
| 14 | GGT | 3.6737 | GLU | 3.5107 | AST | 3.4444 | UKR24 | 2.9771 |
| 15 | ALB24 | 3.5274 | ALB24 | 3.2802 | HT | 3.2875 | MO | 2.9411 |
| 16 | MO | 3.2540 | ER | 3.2712 | AF | 3.2441 | LDC | 2.4165 |
| 17 | LDC | 2.9278 | CA | 3.2323 | CHO | 3.1837 | K | 2.3879 |
| 18 | UZ | 2.9129 | CHO | 3.1705 | GGT | 2.8357 | FOS | 2.3554 |
| 19 | GLU | 2.8069 | K | 3.0896 | ALT | 2.7303 | AST | 2.1649 |
| 20 | CA | 2.7636 | HT | 2.9256 | CA | 2.7213 | ALB24 | 2.1568 |
| 21 | CHO | 2.7498 | UZ | 2.7431 | ALB24 | 2.6150 | GGT | 2.0545 |
| 22 | AST | 2.0089 | LDC | 2.1811 | BA | 2.2721 | GLU | 1.9003 |
| 23 | HDC | 1.8004 | NA | 1.9919 | HB | 1.6473 | UZ | 1.7952 |
| 24 | K | 1.7194 | HB | 1.6115 | NA | 1.5232 | HB | 1.5549 |
| 25 | HB | 1.7003 | AST | 1.6113 | GLU | 1.4462 | NA | 1.1045 |
| 26 | BA | 1.2199 | HDC | 1.4058 | K | 1.0701 | HDC | 0.9180 |
| 27 | BALB | 1.0168 | BA | 0.9584 | HDC | 1.0348 | BALB | 0.7489 |
| 28 | NA | 0.9405 | BALB | 0.8887 | BALB | 0.7876 | BA | 0.5204 |

Source: Authors’ own computation.

**Table 13. Support Vector Machine (SVM) Output for Variable Importance.**

| **A.D.** | **Generalized Anxiety Disorder**  **(GAD)** | | **Agoraphobia**  **(AP)** | | **Panic Disorder**  **(PD)** | | **Social Anxiety Disorder**  **(SAD)** | |
| --- | --- | --- | --- | --- | --- | --- | --- | --- |
| **SN** | **Variable** | **Importance** | **Variable** | **Importance** | **Variable** | **Importance** | **Variable** | **Importance** |
| 1 | EO | 0.0569 | GLU | 0.0661 | EO | 0.0817 | BALB | 0.0798 |
| 2 | BA | 0.0554 | HB | 0.0619 | LY | 0.0681 | TGL | 0.0682 |
| 3 | ER | 0.0553 | GR | 0.0607 | TGL | 0.0581 | HT | 0.0675 |
| 4 | UKR24 | 0.0534 | NA | 0.0593 | AF | 0.0558 | BA | 0.0586 |
| 5 | TGL | 0.0528 | UKR24 | 0.0515 | BA | 0.0551 | LDC | 0.0568 |
| 6 | K | 0.0473 | UR | 0.0499 | UR | 0.0534 | GLU | 0.0527 |
| 7 | TR | 0.0464 | AF | 0.0493 | TR | 0.0485 | UR | 0.0506 |
| 8 | GLU | 0.0451 | CA | 0.0464 | BKR | 0.0415 | TR | 0.0461 |
| 9 | NA | 0.0435 | CHO | 0.0453 | ER | 0.0410 | K | 0.0443 |
| 10 | MO | 0.0430 | BA | 0.0434 | NA | 0.0407 | CA | 0.0423 |
| 11 | HB | 0.0411 | LY | 0.0403 | K | 0.0352 | MO | 0.0389 |
| 12 | LDC | 0.0397 | ER | 0.0380 | HT | 0.0347 | NA | 0.0388 |
| 13 | UR | 0.0388 | TR | 0.0357 | UKR24 | 0.0342 | HDC | 0.0362 |
| 14 | BKR | 0.0386 | EO | 0.0329 | GLU | 0.0330 | AF | 0.0330 |
| 15 | ALB24 | 0.0385 | TGL | 0.0296 | LDC | 0.0315 | LY | 0.0324 |
| 16 | LY | 0.0358 | K | 0.0293 | MO | 0.0309 | FOS | 0.0306 |
| 17 | HT | 0.0332 | AST | 0.0260 | GR | 0.0299 | GR | 0.0273 |
| 18 | GR | 0.0325 | GGT | 0.0256 | ALB24 | 0.0288 | ER | 0.0263 |
| 19 | AST | 0.0278 | BKR | 0.0244 | CA | 0.0284 | UKR24 | 0.0232 |
| 20 | FOS | 0.0256 | LDC | 0.0238 | HB | 0.0250 | EO | 0.0222 |
| 21 | AF | 0.0251 | BALB | 0.0229 | HDC | 0.0236 | GGT | 0.0208 |
| 22 | UZ | 0.0237 | UZ | 0.0226 | CHO | 0.0228 | CHO | 0.0206 |
| 23 | BALB | 0.0228 | HT | 0.0224 | ALT | 0.0214 | ALB24 | 0.0198 |
| 24 | HDC | 0.0199 | ALB24 | 0.0218 | GGT | 0.0164 | HB | 0.0173 |
| 25 | ALT | 0.0165 | HDC | 0.0202 | FOS | 0.0157 | UZ | 0.0164 |
| 26 | CA | 0.0160 | MO | 0.0186 | AST | 0.0157 | ALT | 0.0112 |
| 27 | CHO | 0.0131 | ALT | 0.0165 | BALB | 0.0156 | AST | 0.0100 |
| 28 | GGT | 0.0124 | FOS | 0.0157 | UZ | 0.0132 | BKR | 0.0081 |

Source: Authors’ own computation.

**Table 14. Neural Network (NN) Output for Variable Importance.**

| **A.D.** | **Generalized Anxiety Disorder**  **(GAD)** | | **Agoraphobia**  **(AP)** | | **Panic Disorder**  **(PD)** | | **Social Anxiety Disorder**  **(SAD)** | |
| --- | --- | --- | --- | --- | --- | --- | --- | --- |
| **SN** | **Variable** | **Weights** | **Variable** | **Weights** | **Variable** | **Weights** | **Variable** | **Weights** |
| 1 | AF | 0.1 | CHO | 0.7000 | LDC | 0.4000 | AST | 0.8800 |
| 2 | ALT | -0.06 | AF | 0.2900 | TGL | 0.3700 | CHO | 0.6100 |
| 3 | AST | -0.01 | GLU | 0.2600 | FOS | 0.3500 | ALB24 | 0.4200 |
| 4 | BA | 0.07 | CA | 0.2400 | BA | 0.2700 | BKR | 0.4000 |
| 5 | BALB | 0.03 | FOS | 0.1600 | GR | 0.2500 | UR | 0.3500 |
| 6 | BKR | -0.35 | HT | 0.1500 | LY | 0.1800 | AF | 0.3400 |
| 7 | CA | 0.01 | TR | 0.1500 | HB | 0.1700 | ER | 0.2200 |
| 8 | CHO | -0.57 | GGT | 0.1100 | BALB | 0.1600 | UZ | 0.2100 |
| 9 | EO | -0.01 | MO | 0.0900 | TR | 0.1600 | HT | 0.2000 |
| 10 | ER | -0.37 | AST | 0.0700 | ALT | 0.1100 | UKR24 | 0.1900 |
| 11 | FOS | 0.16 | HB | 0.0700 | GGT | 0.1100 | ALT | 0.1100 |
| 12 | GGT | 0.18 | TGL | 0.0700 | AF | 0.0900 | EO | 0.1100 |
| 13 | GLU | -0.06 | ALB24 | 0.0600 | HT | 0.0900 | BA | 0.0900 |
| 14 | GR | 0.26 | NA | 0.0500 | ALB24 | 0.0400 | GLU | 0.0000 |
| 15 | HB | -0.25 | GR | 0.0400 | CA | -0.0200 | HDC | 0.0000 |
| 16 | HDC | -0.03 | LY | 0.0400 | K | -0.0500 | CA | -0.0400 |
| 17 | HT | 0.42 | UKR24 | 0.0000 | AST | -0.1100 | FOS | -0.0400 |
| 18 | K | 0.02 | EO | -0.0100 | EO | -0.1200 | MO | -0.0400 |
| 19 | LDC | 0.54 | K | -0.0100 | GLU | -0.1500 | NA | -0.0400 |
| 20 | LY | 0.29 | BA | -0.0300 | HDC | -0.1500 | TR | -0.0500 |
| 21 | MO | -0.27 | UZ | -0.0700 | UKR24 | -0.1600 | K | -0.0800 |
| 22 | NA | -0.05 | BALB | -0.1300 | NA | -0.1800 | HB | -0.1300 |
| 23 | TGL | 0.16 | UR | -0.1900 | MO | -0.2100 | GR | -0.1600 |
| 24 | TR | 0.09 | HDC | -0.2900 | BKR | -0.3300 | LY | -0.2600 |
| 25 | UR | -0.31 | ALT | -0.4200 | CHO | -0.3400 | GGT | -0.2900 |
| 26 | UZ | -0.09 | ER | -0.5000 | ER | -0.3600 | BALB | -0.3800 |
| 27 | ALB24 | 0 | LDC | -0.5700 | UZ | -0.4700 | TGL | -0.5400 |
| 28 | UKR24 | 0.22 | BKR | -0.7000 | UR | -0.5000 | LDC | -0.5900 |

Source: Authors’ own computation.
