# Supplementary material for: Understanding importance of clinical biomarkers for diagnosis of anxiety disorders using machine learning models
Source: PLoS One. 2021 May 10;16(5):e0251365. doi: 10.1371/journal.pone.0251365 (PMC8109802; doi:10.1371/journal.pone.0251365)
Supplement: S2 Appendix — (DOCX) [file pone.0251365.s002.docx]

**S2 Appendix**

**Machine Learning Models: Default Settings**

In this study; GLM, Random Forest, Support Vector Machine, Gradient Boosting Machine and Neural Net machine learning models were used. Following are the hyperparameters used with each type of machine learning model:

1. Generalized Linear Model (GLM)

Model link function: family=”binomial”

Prior Weights: weights=NULL

Subsetting the observations: subset=NULL

Starting values for the parameters in the linear predictors: start=NULL

Starting values for the linear predictor: etastart=NULL

Starting values for the vector of means: mustart=NULL

A priori known component included in the linear predictor during fitting: offset=NULL

Auxiliary function for glm fitting: glm.control (epsilon=1e-8, maxit=25)

Method used in fitting the model: method=glm.fit

Number of iterations: iter=25

1. Random Forest Model (RF)

Number of trees: ntree=500

Number of variables tried at each split: mtry=9

1. Support Vector Machine (SVM)

Type of Classification: SVM-Type=C-classification

Type of Kernel: SVM-Kernel=radial

Cost function: cost=1

1. Gradient Boosting Machine (GBM)

Distribution Type: distribution=”bernoulli”

Number of Trees: ntree=1000

Interaction Depth: interaction.depth = 1,

Minimum number of observations in a node: n.minobsinnode = 10,

Shrinkage: shrinkage = 0.1,

Bag Fraction: bag.fraction = 0.5,

Train Fraction: train.fraction = 1,

Cross Validation Folds: cv.folds = 0

1. Neural Net Model

Cross Validation: Method=”repeatedcv”

Repeats: Repeats=10
